# The Serum Level of Fibroblast Growth Factor-23 and Calcium-Phosphate Homeostasis in Obese Perimenopausal Women

**DOI:** 10.1155/2011/707126

**Published:** 2011-11-24

**Authors:** M. Holecki, J. Chudek, A. Więcek, M. Titz-Bober, J. Duława

**Affiliations:** ^1^Department of Internal Medicine and Metabolic Diseases, Medical University of Silesia, ul. Ziołowa 45/47, 40-635 Katowice, Poland; ^2^Department of Pathophysiology, Medical University of Silesia, ul. Medyków 18, 40-752 Katowice, Poland; ^3^Department of Nephrology, Endocrinology and Metabolic Diseases, Medical University of Silesia, ul. Francuska 20-24, 40-027 Katowice, Poland

## Abstract

Plasma FGF-23 concentrations and its relationship with calcium-phosphate homeostasis were evaluated in 48 perimenopausal obese women and in 29 nonobese controls. Serum parathyroid hormone, 25-hydroxyvitamin D_3_, CTX1, osteocalcin, total calcium, phosphorus, creatinine, and plasma intact FGF-23 concentrations were assessed. DXA of lumbar spine and femoral neck was performed to determine bone mineral density (BMD). Plasma iFGF-23 concentration was significantly higher in obese patients (by 42%) and correlated with age and BMD of proximal femur (*R* = −0.346; *R* = 0.285, resp.) but not with markers of bone turnover. However, serum phosphorus level in obese subjects was significantly lower. iFGF-23 concentration correlated significantly with body mass index (*R* = 0.292) and fat content (*R* = 0.259) in all study subjects. Moreover, a significant correlation between iFGF-23 and iPTH (*R* = 0.254) was found. No correlation between serum phosphorus or eGFR and plasma iFGF-23 and between eGFR and serum phosphorus was found. Elevated serum iFGF-23 concentration may partially explain lower phosphorus levels in the obese and seems not to reflect bone turnover.

## 1. Introduction

Epidemiological studies have suggested a protective effect of obesity on postmenopausal bone loss [1]. In general, obesity in women is associated with lower risk of osteoporosis, which may be due to the beneficial effect of hyperestrogenemia [2]. The obese are characterized by calciotropic hormones disturbances, including lower 25-hydroxyvitamin-D_3_ (25-OH-D_3_) and higher parathyroid hormone (PTH) serum concentrations [3, 4], which increases along with the degree of obesity [5, 6]. Lower 25-OH-D_3_ levels in obese may be explained by decreased exposure to sunlight (limited mobility of some obese) [7], inhibited vitamin D hydroxylation in the liver [8], and probably excessive storage of vitamin D in adipose tissue [6].

The regulation of bone metabolism is complex including hormones and a long list of cytokines and growth factors. Recently, a new hormone involved in calcium-phosphate homeostasis was found—fibroblast growth factor-23 (FGF-23). FGF-23 is a 251-aminoacid residues protein that belongs to FGF family [9]. Fibroblast growth factors regulate the development of subcutaneous white adipose tissue (scWAT); however only five of them (FGF-1, 2, 7, 9 and 18) and not FGF-23 were identified in scWAT [10]. Osteocytes and osteoblast turned out to be the major physiological sources of FGF-23 [11–13]. FGF-23 regulates serum phosphate and 1,25(OH)_2_D_3_ levels by acting on the kidney and intestines. FGF-23 reduces serum phosphorus level by suppressing proximal tubular phosphorus reabsorption (similarly to PTH) and, in indirect way, intestinal phosphorus absorption [14]. FGF-23 also suppresses the expression of 1*α*-hydroxylase and enhances the expression of 24*α*-hydroxylase in the kidney, causing the reduction of serum 1,25(OH)_2_D_3_ level [14]. 

An association of plasma FGF-23 concentration with metabolic syndrome has already been proved [15]; however there are no data evaluating its association with bone metabolism in the obese. The aim of this study was to evaluate plasma FGF-23 concentrations and its relationship with calcium-phosphate homeostasis in obese perimenopausal women.

## 2. Materials and Methods

Forty-eight obese perimenopausal women were enrolled into this study (Table 1). Each woman was diagnosed with simple obesity without concomitant diseases. None of them underwent ovariectomy. All subjects were informed about the nature of the study and signed their informed consent forms. The exclusion criteria included current or recent (preceding 3 months) infections, pharmacotherapy affecting bone metabolism, cigarette smoking, and drinking of more than 2 alcohol beverages a week. The control group consisted of 29 nonobese healthy women (Table 1).

Body mass index (BMI) was calculated based on height and weight measurements. Blood sample was obtained from each subject between 8.00 and 9.00 am, after an overnight fast. Serum creatinine, total calcium, and phosphorus concentrations were measured using the commercially available test kits (Roche, Switzerland). Glomerular filtration rate (eGFR) was estimated using the CKD-EPI formula [16].

Plasma and serum samples for intact fibroblast growth factor 23 (iFGF-23), intact parathyroid hormone (iPTH), 25-OH-D_3_, CTX_1_, and osteocalcin estimations were stored at −80°C until the time of the assay.

### 2.1. Laboratory Measurements

Intact FGF-23 was measured by ELISA (Immutopics, San Clemente, USA). The Electrochemiluminiscent ImmunoAssay (Elecsys, Roche Diagnostics GmbH, Germany) was used for iPTH, osteocalcin, and C-terminal telopeptide of type I collagen (CTX1) assays. 25-(OH)-D_3_ was measured using RIA method (Bio Source-EUROPE S.A, Nivelles, Belgium). The concentrations of total calcium and inorganic phosphorus were assessed using spectrophotometer (Point Scientific Inc. MI, USA).

Body composition was analyzed using bioimpedance method (Bodystat 1500, Bodystat Ltd., Great Britain). Dual energy X-ray absorptiometry (DXA method) of lumbar spine and femoral neck for BMD and BMC determinations was performed using Lunar Prodigy Advance apparatus. 

### 2.2. Statistical Analysis

All values are expressed as means ± 95% confidence intervals. Statistical analysis was performed using the STATISTICA 8.0 PL software. The results were analyzed using the Kolmogorov-Smirnoff test. As iFGF-23 values show marked squvenes, logarithmic transformation was performed for calculation of univariate correlations and multiple regression analyses. Mann-Whitney *U* test was used to compare obese to control group. Correlation coefficients were calculated according to Spearman. Separate multiple regression analyses were performed to explain variability of iFGF-23 concentrations, BMD of proximal femur, and lumbar spine. *P* values <0.05 were considered to be statistically significant.

## 3. Results

Obese subjects were characterized by higher BMC and BMD both in the lumbar spine and femoral neck. There were higher serum levels of iPTH and iFGF-23 (by 42%), but lower of 25-OH-vitamin D3, phosphorus, and osteocalcin in obese women than in controls (Table 1). 

**Figure 1:**
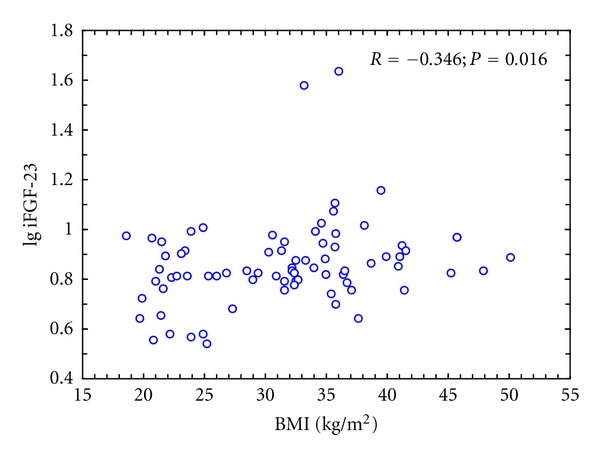
Correlation between BMI and iFGF-23 serum level in obese women and controls.

**Figure 2:**
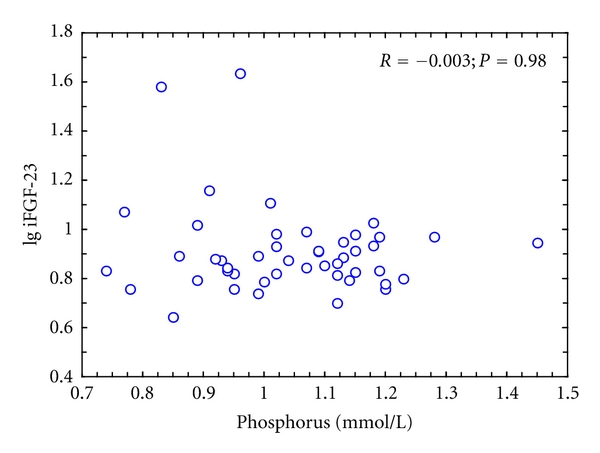
The lack of correlation between serum phosphorus and iFGF-23 concentrations in obese women.

In the combined group of obese and nonobese women iFGF-23 concentration correlated significantly with BMI (*R* = 0.292; *P* = 0.01) (Figure 1), body mass (*R* = 0.241; *P* = 0.035), and fat content (*R* = 0.259; *P* = 0.024). The relationship with waist circumference did not reach the level of statistical significance (*R* = 0.224; *P* = 0.073). There was a correlation between iFGF-23 concentration and proximal femur BMD (*R* = 0.321; *P* = 0.010) but not lumbar spine BMD (*R* = 0.223; *P* = 0.099). Moreover, a significant correlation between iFGF-23 and iPTH (*R* = 0.254; *P* = 0.026) was found. No correlation was demonstrated between serum phosphorus or eGFR and plasma iFGF-23 and between eGFR and serum phosphorus (Figure 2).

In the group of obese women iFGF-23 concentration correlated significantly only with age (*R* = −0.346; *P* = 0.016) and proximal femur BMD (*R* = 0.285; *P* = 0.05). There was also a correlation with eGFR of borderline significance (*R* = 0.266; *P* = 0.07). No significant correlation was found between iFGF-23 plasma concentration and other study parameters in controls. 

Multiple regression analysis did not reveal any independent contribution of iFGF-23 concentrations to both proximal femur and lumbar spine BMD. The variability of proximal femur BMD was in 36.6% explained by age (*β* = −0.391; *P* < 0.001) and BMI (*β* = 0.329; *P* = 0.006). Similarly the variability of lumbar spine BMD was in 17.9% explained by age *β* = −0.354; *P* = 0.007) and serum level of 25(OH)D_3_ (*β* = 0.271; *P* = 0.046).

## 4. Discussion

To the best of our knowledge, this is the first study that evaluated plasma iFGF-23 concentration with reference to bone metabolism in obesity. Our study revealed higher (by 42%) serum level of iFGF-23 in obese individuals compared to nonobese ones. 

A positive correlation between serum level of FGF-23 and BMI, waist circumference, waist-to-hip ratio, and triglycerides has been previously described by Mirza et al. [15]. The authors concluded that higher serum level of iFGF-23 may indicate an increased cardiovascular risk in elderly population; however its direct toxicity has never been shown. The authors did not explain the potential reason of FGF-23 increase in obese individuals. Moreover, the same authors in another study reported an association between elevated serum iFGF-23 concentration and the overall fracture risk in elderly men. This relation remained unaltered after adjustment for BMI, BMD, glomerular filtration rate, 25(OH)_2_D_3_, PTH, and other fracture risk factors [17].

To find out the explanation for our finding we focused on mechanisms potentially influencing serum FGF-23 levels. It has been previously shown that FGF-23 release is regulated by vitamin D_3_, phosphorus, and possibly PTH. Administration of 1,25(OH)_2_D_3_ and dietary phosphorus load was associated with increased FGF-23 levels [18, 19]. It has been demonstrated that PTH stimulates FGF-23 expression, whereas FGF-23 suppresses PTH secretion both *in vitro* and *in vivo *[20–22]. It was suggested that osteoblast stimulation by PTH directly increases skeletal FGF-23 release [19]. However the precise mechanism of this action remains unknown. 

We may suspect that higher level of PTH, previously reported in obese subjects, as well as higher dietary phosphorus may both account for serum FGF-23 increase. However, lower serum phosphorus concentrations in the obese do not support our hypothesis on a compensatory release of FGF-23 in the response to phosphorus accumulation. Perhaps more important than serum phosphorus level is dietary phorphorus intake. As it has been previously shown by Burnett et al., sustained increase in dietary phosphorus was associated with increasing iFGF-23 levels and declining calcitriol levels, while dietary phosphorus restriction reversed these trends [23]. The magnitude of change in serum FGF-23 level with dietary phosphorus depletion was smaller than with dietary phosphorus loading. It suggests that FGF-23 is a hormone that primarily promotes phosphorus wasting [22]. In our study we did not quantify phosphorus content in the diet or its urinary excretion; therefore we cannot analyze the influence of phosphorus consumption on iFGF-23 serum levels. We may suppose that elevated serum FGF-23, observed in obese subjects, may decrease phosphorus levels *via* a direct effect on proximal tubular phosphate reabsorption and/or intestinal phosphorus absorption in addition to glomerular hyperfiltration. The lack of correlation between serum phosphorus and iFGF-23 concentrations and simultaneously an almost significant inverse correlation between eGFR and serum phosphorus seem to support our hypothesis. 

It is well known that obesity is associated with both functional and structural changes within the kidney [24, 25]. Increased GFR in the obese may influence serum phosphorus and even secondarily suppress iFGF-23 secretion.

In the study group of obese women, serum iFGF-23 concentration correlated significantly with age and proximal femur BMD only. Surprisingly, no correlation was found between iFGF-23 and studied markers of bone turnover, and the more so, because the study of Wang et al. showed that FGF-23 appeared to directly inhibit osteoblasts maturation and matrix mineralization [26]. In our study, the osteocalcin serum level was decreased in obese but no correlation with FGF-23 was found. We believe that this finding might be explained by decreased bone turnover in obese individuals, as already described in our previous study [27]. We suspect that the lack of significant correlations may have resulted from a limited number of study subjects. In line with this explanation we have demonstrated a significant, though weak, correlation between iPTH and iFGF-23 concentrations only in the combined group analysis. Recently Marsell et al. have found a positive, yet very weak (*R* = 0.04–0.07), correlation between serum FGF-23 and BMD in elderly men, which disappeared in multivariate correlation adjusting for body weight [28]. This correlation is quite surprising; however, it might be explained by the positive association between FGF-23 and body weight. The authors speculated, that osteocytes have a mechanosensory role, thus increased mechanical load (due to a larger body weight) may stimulate FGF-23 expression. Finally, the authors concluded that FGF-23 is a poor marker of BMD, due to the weak strength of correlation and its dependence on body weight.

Our study has several limitations. The main one, previously mentioned, is the lack of measurement of daily phosphorus consumption and its urinary excretion. Moreover, metabolic and hormonal disturbances found in obese individuals, such as higher serum PTH, eGFR, and lower phosphorus, 25-OH-vitamin D_3_, and osteocalcin levels, may influence iFGF-23 secretion in obese individuals. Given these limitations, our study shows that obese individuals have higher level of iFGF-23 that correlates with proximal femur BMD. However, the mechanism by which iFGF-23 may affect bone metabolism in obese subjects remains unknown. Further studies are necessary to elucidate the cause and role of increased serum iFGF-23 level in the obese.

## 5. Conclusion

Serum levels of iFGF-23 are increased in obese perimenopausal women. This may partially explain lower phosphorus levels in obese subjects and seems not to reflect bone turnover.

## Figures and Tables

**Table 1:** Characteristics of obese women and controls.

	Obese *N* = 48	Controls *N* = 29	
Age (yrs)	55.4 (53.6–57.1)	50.4 (46.0–54.7)	*P* = 0.22
Body mass (kg)	94.5 (90.6–98.3)	62.4 (59.2–65.6)	*P* < 0.001
BMI (kg/m^2^)	36.5 (35.1–37.9)	23.5 (22.4–24.6)	*P* < 0.001
Waist circumference (cm)	106.7 (103.3–110.1)	82.3 (78.3–86.4)	*P* < 0.001
Fat tissue content (%)	49.6 (47.8–51.3)	35.2 (32.3–38.2)	*P* < 0.001
BMD L_2_–L_4_ (g/cm^2^)	1.196 (1.133–1.259)	1.035 (0.930–1.140)	*P* = 0.023
BMD proximal femur (g/cm^2^)	1.026 (0.976–1.076)	0.858 (0.808–0.909)	*P* < 0.001
BMC L_2_–L_4_ (g/cm)	65.0 (60.7–69.3)	56.4 (51.1–61.7)	*P* = 0.036
BMC proximal femur (g/cm)	4.87 (4.58–5.16)	4.23 (3.81–4.64)	*P* = 0.030
Creatinine (mg/dL)	0.64 (0.61–0.67)	0.66 (0.61–0.71)	*P* = 0.77
eGFR (mL/min)	97.4 (94.4–100.3)	99.5 (92.7–106.3)	*P* = 0.46
Total calcium (mmol/L)	2.22 (2.18–2.25)	2.19 (2.13–2.25)	*P* = 0.52
Phosphorus (mmol/L)	1.04 (1.00–1.08)	1.15 (1.09–1.21)	*P* = 0.003
25(OH)D_3_ (ng/mL)	30.2 (26.2–34.2)	43.5 (34.8–52.1)	*P* = 0.016
Osteocalcin (ng/mL)	19.1 (17.4–20.9)	24.0 (20.4–27.6)	*P* = 0.026
iPTH (pg/mL)	47.3 (42.2–52.3)	35.4 (30.4–40.4)	*P* = 0.002
CTX_1_ (ng/mL)	0.24 (0.21–0.27)	0.28 (0.21–0.35)	*P* = 0.73
iFGF-23 (ng/mL)	9.14 (7.12–11.15)	6.44 (5.70–7.18)	*P* = 0.005

PTH: parathormone; CTX_1_: C-terminal telopeptide of collagen type I; BMI: body mass, BMD: bone mineral density, BMC: bone mineral content, iFGF-23: intact fibroblast growth factor 23, eGFR: estimated glomerular filtration rate.
